# Biochemical and *Hsp70* gene expression changes in *Apis mellifera* workers following water and food deprivation

**DOI:** 10.1186/s40850-026-00265-3

**Published:** 2026-05-13

**Authors:** Amr M. A. Mohamed, Eslam M. Omar, Ebrahim M. E. Alhousini

**Affiliations:** 1 Department of Zoology, Faculty of Science, Qena University, Qena, 83523 Egypt; 2 Plant Protection Department, Faculty of Agriculture, Assiut University, Assiut, 71526 Egypt

**Keywords:** Honeybee, Dehydration, Starvation, Enzyme activity, *Hsp70* gene

## Abstract

**Background:**

Resource scarcity poses a real challenge to living organisms. The present study aimed to understand the implications of water and food lack on some biochemical markers. The study also aimed to investigate the expression of *Hsp70* gene under the same stress conditions. The *Hsp70* gene maintains cellular proteostasis. It is unique in its high sensitivity and rapid response to various stress factors compared to other *Hsps*.

**Methods:**

Brood frames of Carniolan hybrid bees were incubated at 30 °C and 70% RH. Emerged bees were placed in plastic cages (30 individuals per cage) and received daily diets of pollen-sugar pastes and sugar solutions. After nine days, bees were independently subjected to dehydration and starvation experiments for 24 h. One group was deprived of only sugar solutions (Dehydrated DH), another group was denied both sugar solutions and pollen pastes, receiving only tap water (Starved ST), while a third group continued without water or food deprivation (Control C). Bee samples were collected at 12-hour intervals in each experiment for subsequent investigations. Biochemical measurements were performed on total protein, total antioxidant capacity, peroxidase, catalase, glutathione S-transferase, and acid phosphatase. Gene expression of *Hsp70* gene was under observation. Bee weights were also considered.

**Results:**

Both dehydrated and starved bees showed notable changes in their examined biomarkers. Starvation had a more pronounced and rapid biochemical effect. The decreases in *Hsp70* mRNA levels following water or food deprivation were surprising to us and reflect the severity of these two stressors. Other differences were also recorded in the weights of dehydrated and starved bees.

**Conclusions:**

This study reveals honeybees’ extreme sensitivity to nutritional stress and warns against depriving them of water or food for even a few hours.

**Supplementary Information:**

The online version contains supplementary material available at 10.1186/s40850-026-00265-3.

## Background

Biochemical and molecular changes in stressed honeybees (*Apis mellifera*) remain a topic of research interest. Many studies have discussed these changes under the influence of different stress factors such as chemical stress caused by insecticides and environmental stress caused by urban beekeeping [1–4]. Surprisingly, little attention has been given to nutritional stress, that threatens bee survival, and addressing it is an urgent matter that cannot be delayed. Little prior research has reported negative signs of immune system sensitivity or mineral metabolism efficiency in honeybees experiencing either dehydration or starvation [5, 6]. The knowledge gap also includes the effects of water and food deprivation on oxidative stress enzymes and *Hsp70* gene (Heat shock protein 70 kDa) that maintains proper folding, stabilization, and quality control of proteins within the cell.

Coping with stressful conditions in an insect requires a high energy cost. This leads to increased mitochondrial respiration, thereby accelerating the electron transport chain (ETC). Under high demand, some electrons leak from the ETC and react with oxygen, forming reactive oxygen species (ROS). These species interact with cellular components and form harmful peroxides. Unfortunately, the inability to neutralize accumulated ROS leads to oxidative stress [7].

Oxidative stress in insects can be evaluated through several biomarkers. Total protein (TP) measurement indicates protein mobilization or degradation under stress conditions [8]. It has been observed that prolonged pollen deprivation may reduce protein concentrations in honeybees [9, 10]. Total antioxidant capacity (TAC) measures the combined activity of all enzymatic and non-enzymatic antioxidant defenses [11]. Interestingly, good nutrition may improve bee immunity and raise TAC levels [12, 13].

The same improvement in the activity of both peroxidase (POD) and catalase (CAT) enzymes can be observed in worker bees consuming high-quality diets [14, 15]. Both enzymes help maintain redox balance in honeybees throughout stressful conditions. POD uses reducing agents such as glutathione (GSH) to detoxify peroxides. CAT breaks down hydrogen peroxide into water and oxygen [11]. Further, the glutathione S-transferase (GST) enzyme conjugates GSH to harmful compounds, such as oxidized lipids, converting them into less toxic and more easily excreted substances [16]. Unfortunately, acute stressors, such as fungicides, may adversely affect GST activity in worker bees [17, 18]. Stressors may cause organelle damage under certain conditions. However, lysosomal activity can help in digesting damaged organelles. This can be considered a cellular repair mechanism mediated by hydrolase enzymes, such as acid phosphatase (ACP) [11].

Stress disrupts protein homeostasis, leading to protein misfolding and aggregation in stressed insects [19]. As a result, the need for heat shock protein (*Hsp*) genes increases. *Hsp* genes encode chaperone proteins that mainly assist in the correct folding of the newly synthesized polypeptides and prevent misfolded proteins from becoming aggregated [20]. Thus, *Hsp* genes can be considered molecular biomarkers of stressors, and investigating alterations in *Hsp* expression levels may enhance our understanding of bee responses to stress at both cellular and physiological levels [21, 22]. Based on the molecular weight of the chaperone proteins they produce, *Hsp* genes are classified into several families, including *Hsp110*, *Hsp90*, *Hsp70*, *Hsp60*, *Hsp40*, and small *Hsps*. The *Hsp70* gene is unique in its high sensitivity and rapid response to various stress factors compared to other *Hsps*. It also plays a central chaperone role under stressful conditions and shows distinct alterations when bees encounter different stressors. *Hsp70* protein binds early to nascent polypeptides or denatured proteins and prevents aggregation [19–23]. Therefore, the gene’s response pattern to various stress factors has been the subject of extensive research [24–26].

Body weight loss is a critical indicator of stress in honeybees, reflecting changes in feeding behavior, nutrient absorption, and overall physiological balance [9, 27]. Recent research has demonstrated that a variety of stressors, such as pollutants and nutritional imbalances, can dramatically reduce honeybee body weight [7, 8, 27]. Stressors can affect key physiological functions in honeybees, which in turn can affect body weight and nutritional status during extended exposure [24, 28]. Furthermore, zinc‑methionine, selenium (Sel‑Plex), thiamethoxam, clothianidin, and imidacloprid insecticides altered flight muscle activity and heat balance, which may indirectly affect energy expenditure and resource intake, highlighting the significance of body weight as an additional sensitive indicator of stress in honeybees [15, 28].

Generally, our study hypothesizes that depriving worker bees of water or food will result in either activation or inhibition in both antioxidant metabolic activity and *Hsp70* gene expression. The severity and patterns of these biochemical and molecular changes will be investigated and discussed 12 and 24 h after exposure to the stressors. This will help us better understand the roles of the *Hsp70* gene as a genetic marker and specific antioxidant enzymes in bees during water or food scarcity.

## Materials and methods

### Experimental design

Brood frames of Carniolan hybrid bees were brought from the South Valley University apiary to the Entomology Lab, Faculty of Science, South Valley University, Egypt. The frames were incubated at 30 °C and 70% RH in an incubator (Acculab GRC-450 F, USA) [29]. The bees began to emerge within 24 h of the brood frames being placed in the incubator. Under the same conditions, emerged adults were placed in 250 ml plastic cages (30 individuals per cage; 3 cages per group; 3 groups) (Figure S1).

The experimental groups were designated as Control (C), dehydrated (DH) and starved (ST). For nine days, each cage received a daily diet of 0.5 g of pollen-sugar paste (1:9) with 9% moisture content and 1 ml of 50% sucrose solution (w/v) [30]. The pastes were prepared from ground pollen and powdered sugar. The pollen mainly comes from alfalfa, date palm, jujube and lemon. These are rich in proteins, amino acids, carbohydrates, lipids, minerals, vitamins, and bioactive compounds like phenolics and flavonoids. The sugar used was cane sugar. The source of feed was uniform throughout the experiment period and it was replaced every day. After day 9, the experiment paths changed for the following 24 h. Group DH was deprived of only sugar solutions, whereas Group ST was denied both sugar solutions and pollen pastes, receiving only 1 ml of tap water per cage. Group C continued until the end of the experiment without water or food deprivation. Bee samples were collected at 12-hour intervals in each experiment for subsequent investigations. The time points investigated in this study (12 and 24 h) were determined based on preliminary experiments that revealed early biochemical changes in the dehydrated and starved bees.

### Biochemical investigation

Following the experiment, C, DH, and ST samples were maintained at -20 °C for subsequent biochemical analyses. Each biomarker analysis was performed on whole bees, with 3 bees per group per time point. The samples were thereafter homogenized in distilled water (50 mg /1 ml) using a chilled glass Teflon tissue homogenizer (Mechanic-Preczyina, Poland) and centrifuged (8000 rpm, 15 min. at 2 °C). Their extracted supernatants were then used in later analyses [31]. A double-beam ultraviolet/visible spectrophotometer (Spectronic 1201, Milton Roy Co., USA) was used to measure absorbance for each biochemical marker. Total protein (TP) concentration was quantified using Coomassie Brilliant Blue reagent (Sigma-Aldrich Chemicals, St. Louis, MO, USA), and absorbance was read at 595 nm [32]. Total antioxidant capacity (TAC) was estimated using spectral analysis of phosphomolybdenum complex (Stanbio laboratory, Texas, USA) at 695 nm [33]. Peroxidase (POD) activity was measured based on the oxidation of guaiacol (Stanbio laboratory, Texas, USA), and absorbance was read at 470 nm [34]. Catalase (CAT) activity was evaluated employing hydrogen peroxide (Sigma-Aldrich Chemicals, St. Louis, MO, USA) as the substrate, and absorbance was recorded at 510 nm [35]. Glutathione S-transferase (GST) level was assessed via the enzyme-catalyzed conjugation of reduced glutathione GSH to 1-chloro-2, 4-dinitrobenzene (CDNB) (BDH Chemicals Ltd., Poole, England), with the increase in absorbance monitored at 340 nm [36]. Acid phosphatase (ACP) activity was estimated using disodium phenylphosphate substrate (Ubichem Ltd., Hampshire, England), and light absorbance was detected at 510 nm [37].

### Gene expression analysis

#### Total RNA extraction and cDNA synthesis

Molecular investigations were performed in Genetics and Molecular Biology Lab of Zoology Department, Faculty of Science, Qena University, Egypt. Nine bee samples of each group; C, DH and ST were ground, homogenized, and their RNA was extracted. Extraction was performed using the ABT Total and Micro RNA Mini Kit (Applied Biotechnology, Egypt). Using a NanoDrop™ LITE Spectrophotometer (Thermo Scientific, USA), RNA concentration and purity were evaluated. Reverse transcription was performed with 1 µg of total RNA (purity 2.1 ± 0.2) using the ABT 2x RT Mix cDNA synthesis kit (Applied Biotechnology, Egypt) according to the manufacturer’s instructions.

#### Quantitative real-time PCR (qRT-PCR)

Synthesized cDNA samples were subjected to qRT-PCR using CFX 96TM Real-Time PCR detection equipment (BIO RAD T100™, USA) and the ABT 2x qRT-PCR SYBR^®^ Green (Applied Biotechnology, Egypt). The reaction consisted of 10 µL of 2× SYBR^®^ Green, 1 µL of each primer for the target gene, and 2 µL of cDNA template, adjusted to a final volume of 20 µL with nuclease-free water (Qiagen, Germany). The thermal cycling protocol comprised an initial denaturation at 95 °C for 10 min, followed by 40 cycles of 95 °C for 30 s, 60 °C for 1 min, and a final extension at 95 °C for 10 s. The temperature range for the melting curve analysis was set from 65 ℃ to 95 ℃, increasing by 0.5 ℃ every 0.05 s. The primers used were purchased from Macrogen, Korea, and are listed in Table 1. The cycle threshold (Ct) value was also determined. Samples were analyzed in triplicate. Gene expression was quantified for each group using the comparative Ct ($\:{2}^{-\varDelta\:\varDelta\:Ct}$) method [38], with *Hsp70* gene expression normalized to *β-Actin* as a reference gene [39].

#### Fresh weight calculation

Fresh weight average of C, DH, and ST bees were identified using a sensitive four-digit balance (RADWAG AS 220/C/1, 220 g capacity, 0.1 mg readability, Poland) throughout 24 h of the experiment (*n* = 10 bees per group per time point).

### Data analysis

GraphPad Prism version 8.0.2 software was used in data analysis. Biochemical parameters (*n* = 3 per group per time point), *Hsp70* gene expression levels (*n* = 9 per group per time point), and body weights (*n* = 10 per group per time point) were assessed separately. All data passed normality verification. This was done using the Shapiro-Wilk and D’Agostino-Pearson tests. Accordingly, Post hoc pairwise comparisons using Tukey’s test (*P* ≤ 0.05) were conducted after two-way ANOVA (α = 0.05). The two-way ANOVA allowed us to examine the effects of group factor (nutritional condition), time factor, and their interaction, while the post hoc Tukey’s test enabled comparison of differences between groups at each time point.

**Table 1:** Primers used in qRT-PCR

Target gene	Accession No.	Primer Sequence	Amplicon Size (bp)	Reference
*Hsp70*	NM_001160072.1	F: ATCAACCTGGCGTCTTGATTC R: TGAGGTACACCTCTAGGTGC	118	[40]
*β-Actin*	AB023025.1	F: CCATGTATCCTGGAATCGCAG R: AGAAGCAAGAATTGACCCACC	134	[23]

## Results

### Biomarkers

#### Total protein (TP)

**Fig. 1:**
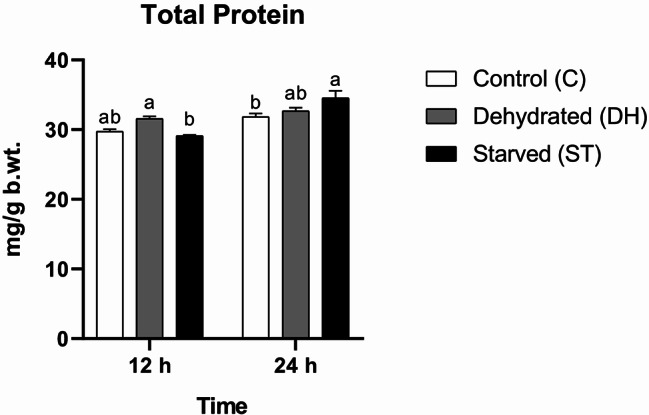
Means of total protein (TP) concentrations (± SEMs) in 9-day-old control (C), dehydrated (DH) and starved (ST) *Apis mellifera* workers after 12 and 24 h of the experiment. Different letters show statistical significant differences (*p* < 0.05)

The differences in total proteins were more pronounced between the two time points (F = 42.17, df = 1, *P* < 0.0001) than between the treatments (F = 3.358, df = 2, *P* = 0.0695). The interaction term was statistically significant (F = 8.572, df = 2, *P* = 0.0049). Biologically, this suggests that the responses of bees to the stress factors were not uniform over time, meaning the effect of the each stress differed between the two time points. Post-hoc multiple comparisons reported only one statistical significant difference in favor of DH versus ST bees after 12 h of the experiment (C = 29.73 ± 0.33 mg/g body weight (mg/g b.wt.), DH = 31.57 ± 0.35 mg/g b.wt., ST = 29.1 ± 0.15 mg/g b.wt.). The next 12 h brought another statistical significant increase in TP content, but this time it was in ST bees relative to controls (C = 31.83 ± 0.5 mg/g b.wt., DH = 32.7 ± 0.46 mg/g b.wt., ST = 34.53 ± 1.03 mg/g b.wt.) (Fig. 1).

#### Total antioxidant capacity (TAC)

**Fig. 2:**
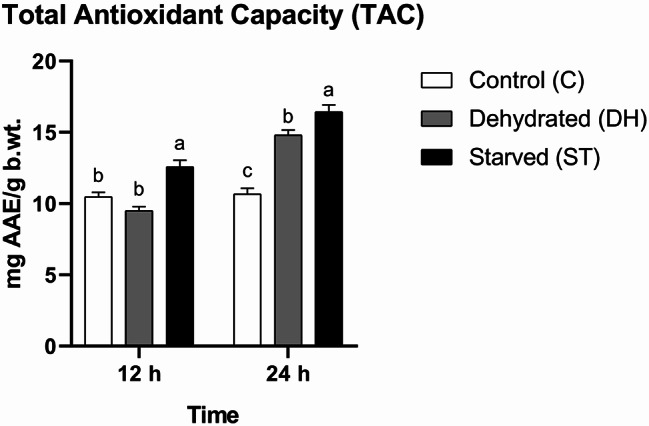
Means of total antioxidant capacity (TAC) (± SEMs) in 9-day-old control (C), dehydrated (DH) and starved (ST) *Apis mellifera* workers after 12 and 24 h of the experiment. Different letters show statistical significant differences (*p* < 0.05)

The main impacts of time (F = 93.89, df = 1, *P* < 0.0001), and nutritional condition (F = 50.3, df = 2, *P* < 0.0001) on TAC differences were statistically significant. Additionally, their interaction was also statistically significant (F = 22.21, df = 2, *P* < 0.0001). TAC varied over time and differed among nutritional conditions, with each stress factor producing a distinct time-dependent response. The need for free radical scavenging was statistically significantly higher in Group ST compared to Groups DH and C after the first 12 h of the experiment. No statistical significant variations were observed between Groups C and DH (C = 10.47 ± 0.32 mg AAE/g b.wt., DH = 9.5 ± 0.29 mg AAE/g b.wt., ST = 12.57 ± 0.47 mg AAE/g b.wt.). Group ST maintained the highest TAC mean until the end of the experiment, followed by Groups DH and C, respectively, in a declining trend. Statistical significant differences were recorded among the three groups (C = 10.67 ± 0.41 mg AAE/g b.wt., DH = 14.8 ± 0.36 mg AAE/g b.wt., ST = 16.43 ± 0.48 mg AAE/g b.wt.) (Fig. 2).

#### Peroxidase (POD)

**Fig. 3:**
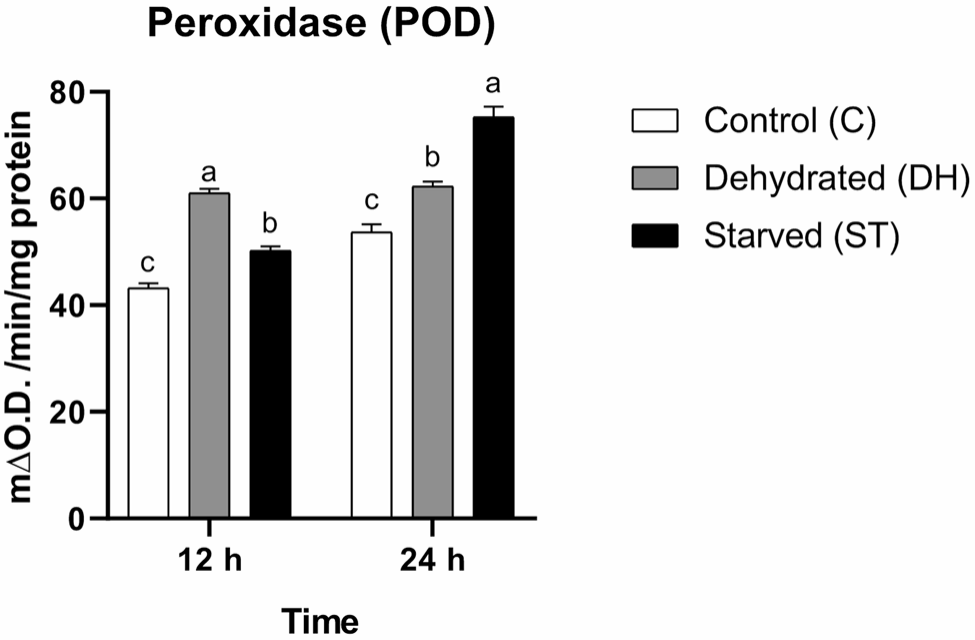
Means of peroxidase (POD) activity rates (± SEMs) in 9-day-old control (C), dehydrated (DH) and starved (ST) *Apis mellifera* workers after 12 and 24 h of the experiment. Different letters show statistical significant differences (*p* < 0.05)

Findings detected statistical significant main influences of time and nutritional condition, as well as a statistical significant interaction effect on POD activity (time: F = 153.4, df = 1, *P* < 0.0001; group: F = 86.21, df = 2, *P* < 0.0001; interaction: F = 48.85, df = 2, *P* < 0.0001). This suggests that both stress factors differentially influenced POD activity across the two time points. Tukey’s post hoc analysis indicated that dehydration and starvation stimulate peroxidase activity. At both time points, DH and ST bees had significantly higher means than C bees, and statistical significant differences were observed among all groups at each time point. Notably, DH bees showed the highest mean at the first 12-h period (C = 43.23 ± 0.85 m∆O.D./min/mg protein, DH = 61 ± 0.85 m∆O.D./min/mg protein, ST = 50.23 ± 0.79 m∆O.D./min/mg protein). In contrast, ST bees exhibited the highest mean at the second 12-h period (C = 53.67 ± 1.52 m∆O.D./min/mg protein, DH = 62.3 ± 0.89 m∆O.D./min/mg protein, ST = 75.3 ± 1.91 m∆O.D./min/mg protein) (Fig. 3).

#### Catalase (CAT)

**Fig. 4:**
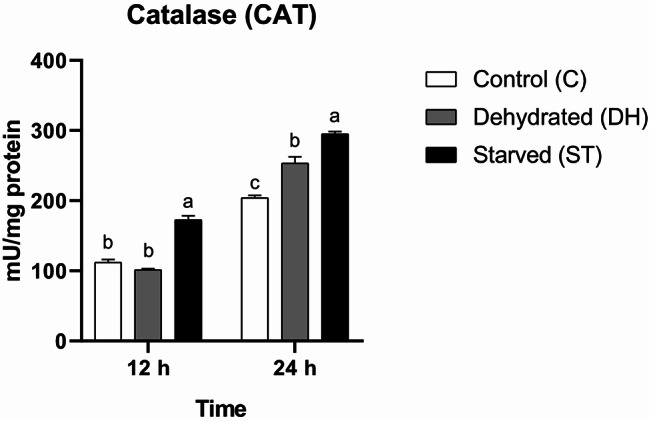
Means of catalase (CAT) activity rates (± SEMs) in 9-day-old control (C), dehydrated (DH) and starved (ST) *Apis mellifera* workers after 12 and 24 h of the experiment. Different letters show statistical significant differences (*p* < 0.05)

The change in CAT activity was statistically significantly affected by time (F = 808.4, df = 1, *P* < 0.0001), nutritional condition (F = 112.2, df = 2, *P* < 0.0001), and their interaction (F = 16.27, df = 2, *P* = 0.0004). The measured CAT exhibited temporal variation and differed among treatments. A 12-h starvation period was sufficient to induce a statistical significant increase in enzyme activity in Group ST compared with the other groups. No statistical significant differences were noticed between Groups C and DH (C = 112 ± 4.16 mU/mg protein, DH = 101.33 ± 1.86 mU/mg protein, ST = 172.67 ± 5.9 mU/mg protein). At the end of the experiment, the CAT activity levels in all groups were generally elevated. However, each group exhibited a statistically significantly distinct value, with Group ST having the highest, Group DH intermediate, and Group C the lowest measurement (C = 204 ± 3.51 mU/mg protein, DH = 253.33 ± 9.28 mU/mg protein, ST = 295 ± 3.46 mU/mg protein) (Fig. 4).

#### Glutathione S-Transferase (GST)

**Fig. 5:**
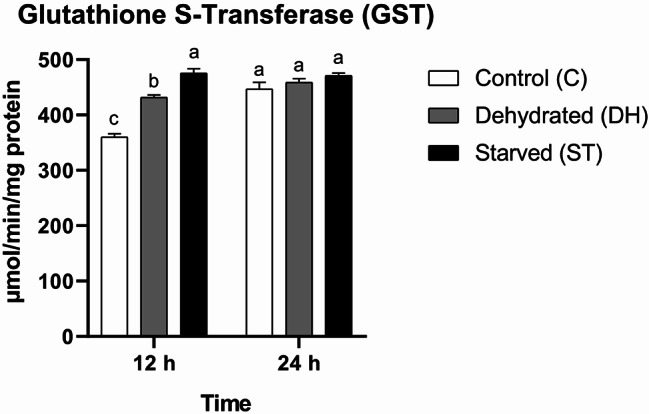
Means of Glutathione S-Transferase (GST) activity rates (± SEMs) in 9-day-old control (C), dehydrated (DH) and starved (ST) *Apis mellifera* workers after 12 and 24 h of the experiment. Different letters show statistical significant differences (*p* < 0.05)

The statistical significant differences in GST activity were attributed to both factors (time and nutritional condition) and their interplay (time: F = 37.05, df = 1, *P* < 0.0001; nutritional condition: F = 45.7, df = 2, *P* < 0.0001; interaction: F = 19.97, df = 2, *P* = 0.0002). GST showed time-dependent variation and differed between treatments. ST bees recorded the highest GST activity at the mid-point of the experiment, followed by DH and C bees, respectively, in descending order, with statistical significant variations observed between all groups (C = 360.33 ± 5.78 µmol/min/mg protein, DH = 431.67 ± 4.41 µmol/min/mg protein, ST = 475.67 ± 7.88 µmol/min/mg protein). After 24 h, no statistical significant differences were reported among all groups (C = 447 ± 11.93 µmol/min/mg protein, DH = 459 ± 6.66 µmol/min/mg protein, ST = 471 ± 4.58 µmol/min/mg protein) (Fig. 5).

#### Acid phosphatase (ACP)

**Fig. 6:**
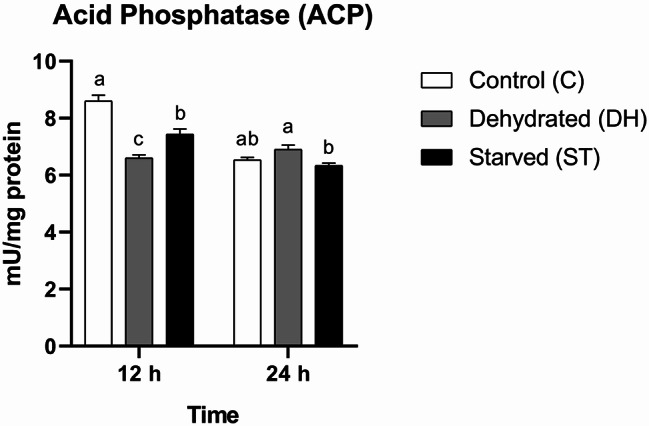
Means of acid phosphatase activity (ACP) rates (± SEMs) in 9-day-old control (C), dehydrated (DH) and starved (ST) *Apis mellifera* workers after 12 and 24 h of the experiment. Different letters show statistical significant differences (*p* < 0.05)

ACP activity was statistically significantly modulated by the individual effects of time (F = 63.21, df = 1, *P* < 0.0001) and nutritional condition (F = 17.72, df = 2, *P* = 0.0003), as well as by their combined interaction (F = 32.68, df = 2, *P* < 0.0001). This suggests that both stressors differentially influenced ACP activity across the two time points. After 12 h of the experiment, acid phosphatase activity differed significantly among all groups, with Group C exhibiting the highest value, followed by Group ST, while Group DH showed the lowest (C = 8.6 ± 0.21 mU/mg protein, DH = 6.6 ± 0.12 mU/mg protein, ST = 7.43 ± 0.19 mU/mg protein). The subsequent 12-h period brought another dramatic change in ACP activity between groups. Group ST showed the lowest mean activity, which did not differ significantly from Group C but differed significantly from Group DH. No statistical significant difference was found between Groups C and DH (C = 6.53 ± 0.09 mU/mg protein, DH = 6.9 ± 0.15 mU/mg protein, ST = 6.33 ± 0.09 mU/mg protein) (Fig. 6).

#### Gene expression

**Fig. 7:**
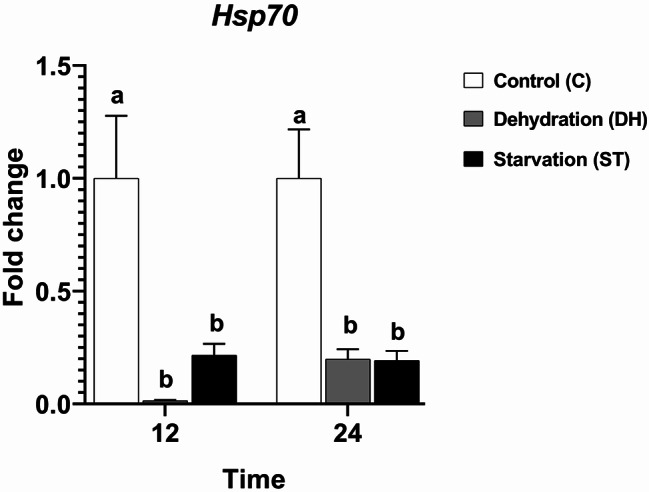
Transcriptional levels of the *Hsp70* gene (± SEMs) in 9-day-old control (C), dehydrated (DH) and starved (ST) *Apis mellifera* workers after 12 and 24 h of the experiment. Different letters show statistical significant differences (*p* < 0.05)

Nutritional condition (stress or control) was the sole statistical significant determinant of changes in *Hsp70* gene expression (F = 22.21, df = 2, *P* < 0.0001), with neither time (F = 0.1989, df = 1, *P* = 0.6636) nor the interaction between time and condition (F = 0.2962, df = 2, *P* = 0.7489) exerting a statistically significant effect. Tukey’s post-hoc test revealed a consistent pattern at both time points, with Groups DH and ST showing statistically significant lower *Hsp70* gene expression fold change compared to Group C. Groups DH and ST did not differ significantly at the two recorded time points (12 h: C = 1 ± 0.28, DH = 0.01 ± 0.0037, ST = 0.22 ± 0.05; 24 h: C = 1 ± 0.22, DH = 0.2 ± 0.04, ST = 0.19 ± 0.04) (Fig. 7).

#### Fresh weight

**Fig. 8:**
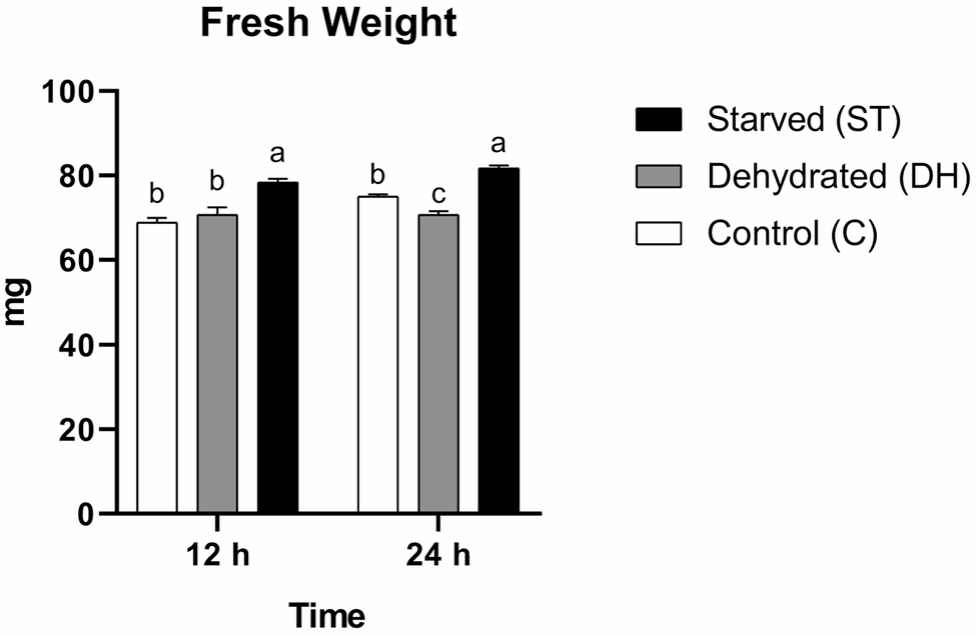
Means of fresh weights (± SEMs) of 9-day-old control (C), dehydrated (DH) and starved (ST) *Apis mellifera* workers after 12 and 24 h of the experiment. Different letters show statistical significant differences (*p* < 0.05)

The changes in fresh weight were significantly affected by time (F = 13.59, df = 1, *P* = 0.0031), nutritional condition (F = 46.4, df = 2, *P* < 0.0001), and their interaction (F = 4.305, df = 2, *P* = 0.039). Fresh weight average fluctuated over time and varied among treatments, with each stressor producing a unique time-dependent pattern. Such changes were observed only during the first 12 h due to food deprivation. Group ST, which attained the highest mean value, exhibited statistically significant differences from both Groups C and DH, whereas no statistical significant variation was observed between Groups C and DH (C = 68.83 ± 1.09 mg, DH = 70.67 ± 1.76 mg, ST = 78.33 ± 0.88 mg). Over the subsequent 12 h, statistical significant differences were observed among all groups. Group ST maintained the highest mean, followed by Group C, while Group DH showed the lowest (C = 75 ± 0.58 mg, DH = 70.67 ± 0.88 mg, ST = 81.67 ± 0.67 mg) (Fig. 8).

## Discussion

Metabolic activity in an organism is a mirror of its health. It is affected by various internal and external factors, including nutrition. In honeybees, variations in nutritional condition are closely linked to shifts in biomarker profiles, reflecting underlying metabolic adjustments [41].

One of our striking observations was the statistical significant increase in Total protein (TP) after a full day of food deprivation in worker bees. This can be understood in the context of the insect mobilizing internal reserves to maintain protein synthesis during starvation [42, 43]. However, the prolonged exposure to different stressors may lead to adverse effects and reduce protein levels. Pollen deprivation for long periods, starting from 7 days, leads to statistical significant decreases in protein concentrations in worker bees [9, 10]. Similarly, chronic exposure to fungicides may reduce protein levels [44].

Total antioxidant capacity (TAC) can increase in honey bees in response to two distinct and unrelated factors: taking a high-quality diet [12, 13], and exposure to stress, such as insecticides [1, 45]. The underlying causes are fundamentally different: one reflects immune enhancement, while the other signals a compensatory response to stress. Our relevant findings revealed that both dehydration and starvation induced TAC elevations in worker bees. Nevertheless, the response was faster under starvation stress.

Either dehydration or starvation causes a statistically significant increase in peroxidase (POD) enzyme activity in honeybees, according to our results. Exposure to heavy metals and pesticides may also activate peroxidases [46–48]. However, adequate protein nutrition can alleviate the effects of oxidative stress and reduce the need for these enzymes’ activity [47]. Monofloral pollen diets from plants such as *Phacelia*, rapeseed, buckwheat, and goldenrod can enhance the performance of peroxidases in monoculture conditions [14].

Catalase (CAT) differs from peroxidases in that it breaks down hydrogen peroxide directly and does not require a reducing agent [11]. Nutrition’s effects on CAT activity remain a topic of interest in bee research. Food fortified with organic zinc or probiotic microorganisms might improve CAT levels [49, 15]. Our study shows that the complete absence of food triggers a defensive response in CAT and elevates it to statistical significant levels. Water deprivation has the same effect at a slower pace. Insecticide-induced stress may also stimulate CAT [50]. However, this enzyme might be depleted under the acute effect of simultaneous use of tebuconazole and fluopyram fungicides [4].

Statistical dramatic alterations in glutathione S-transferase (GST) activity in both dehydrated and starved bees caught our attention; a rapid active response followed by a return to standard rates. It has been shown that GST level may be inversely proportional to stress intensity [2, 17, 18]. This can be interpreted in terms of energy trade-offs in insects. Suppression of certain enzymes in favor of others at a specific time point may occur during acute and prolonged stress [8, 51].

Temporary inhibitions in acid phosphatase (ACP) were noticed in worker bees following water and food deprivation. The subsequent regular activity suggests enzyme recovery. This is a part of insect adaptation mechanisms [11]. Comparable patterns of ACP activity in stressed insects were reported [52, 53].

Honey bees may experience expressional changes in *Hsp70* gene due to seasonal climatic fluctuation [3, 54], geographic location [55, 56], temperature [57, 24], insecticide application [58, 26], population density [59], electromagnetic radiation [25], UV irradiation [40], cage rearing conditions [60], and finally, nutritional condition [23, 61, 62]. Our study added a new, amazing dimension to the effect of nutritional conditions on worker bees. The complete absence of either water or food resulted in a statistical significant downregulation of *Hsp70* gene expression. This might be understood in terms of *Hsp70‑HSF1* autoinhibitory feedback. The *Hsp70* protein binds to the transcription factor *HSF1*, stopping it from activating the *Hsp70* gene again. This mechanism might occur during acute stress to conserve energy and prevent potential false interference with non-target proteins [21]. Our study examined *Hsp70* gene expression in the whole bee body, whereas a previous one focused on investigating the *Hsp70* gene expression only in the heads of dehydrated and starved bees [40]. Both studies emphasize the potential difference in gene expression between the tissue and the whole body under a given stress. Inhibition of *Hsp70* gene expression in response to severe or prolonged stressful conditions has been reported in several insect species [63–65].

Given the changes in the fresh weights of stressed worker bees, we have obtained surprising results. Dehydration led to a gradual weight loss, whereas starvation led to a rapid and persistent weight gain. The most plausible interpretation is that the water content decreased in dehydrated bees and increased in starved bees. Food deprivation might have driven worker bees to drink water voraciously in search of any source of energy. In a comparable context, bees meet their energy needs and instinctively consume excess honey to cope with harsh winter conditions [66]. Recent studies have shown that the pattern of bee weight responses to different stressors as microplastics, insecticides, and ambient temperatures is not uniform [27, 28].

Overall, we can say that resource scarcity poses a serious threat to bees. Water and food deprivation led to statistical significant changes in metabolic activity and *Hsp70* gene expression in bee workers. These observed changes confirmed our previous hypothesis and surprised us with their rapid pace. The results of this study offer a strong foundation for future research.

Our laboratory study was required to isolate variables and establish baseline data. However, the sample size is small and we still need a wider scope in field studies. To build on this data, additional field investigations are recommended to explore the implications of water and food deprivation for bee society across diverse environmental conditions.

## Electronic Supplementary Material

Below is the link to the electronic supplementary material.

Supplementary Material 1

## Data Availability

The datasets and materials of the current study are available from the corresponding author upon request.
