# Supplementary figures and images for: Biochemical and Hsp70 gene expression changes in Apis mellifera workers following water and food deprivation
Source: BMC Zool. 2026 May 13;11:16. doi: 10.1186/s40850-026-00265-3 (PMC13169560; doi:10.1186/s40850-026-00265-3)

| 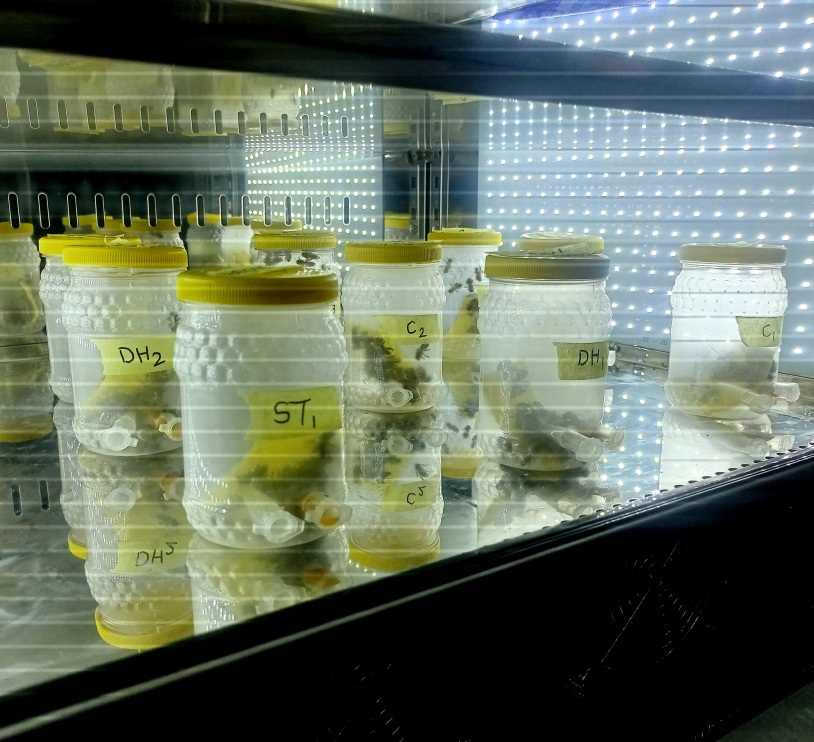 |
| --- |
| **Figure S1:** Plastic rearing cages |

Supplement: Supplementary file 1 — Supplementary Material 1 [file 40850_2026_265_MOESM1_ESM.docx]
